# The effect of D-chiro-inositol on renal protection in diabetic mice

**DOI:** 10.18632/aging.204019

**Published:** 2022-04-19

**Authors:** Chunxue Fan, Dandan Zhang, Junling Zhang, Jinwei Li, Yu Wang, Linghuan Gao, Shuying Han

**Affiliations:** 1 School of Pharmacy, North China University of Science and Technology, Tangshan 063210, Hebei, PR China; 2 Clinical Medical College, North China University of Science and Technology, Tangshan 063210, Hebei, PR China; 3 School of Basic Medical Sciences, North China University of Science and Technology, Tangshan 063210, Hebei, PR China; 4 Hebei Key Laboratory for Chronic Diseases, Tangshan Key Laboratory for Preclinical and Basic Research on Chronic Diseases, School of Basic Medical Sciences, North China University of Science and Technology, Tangshan 063210, Hebei, PR China; 5 School of Nursing and Health, Caofeidian College of Technology, Tangshan 063210, Hebei, PR China

**Keywords:** D-chiro-inositol, diabetic nephropathy, db/db mice, creatinine, serum urea nitrogen

## Abstract

D-Chiro-inositol (DCI) exerts a hypoglycaemic effect, participates in lipid metabolism and reduces kidney damage. In this study, we preliminarily explored the protective effect of DCI on renal injury in diabetic mice. Male db/db mice were used in this study. After treatment with DCI (35 and 70 mg/kg/d) for 6 consecutive weeks, random blood glucose (RBG) measurements were conducted at 0 and 6 weeks. Creatinine (Cr) and serum blood urea nitrogen (BUN) levels were measured using assay kit, and morphological changes in the kidneys were observed by HE staining, Masson staining and electron microscopy. Immunohistochemical and Western blot experiments were used to examine the protein expression of matrix metalloproteinase-9 (MMP-9), nuclear factor-κB (NF-κB) and peroxisome proliferator-activated receptor-γ (PPAR-γ). We discovered that the increased RBG levels were alleviated after treatment with DCI. Moreover, the Cr and BUN levels were reduced, glomerular mesangial hyperplasia was alleviated, and the degree of renal fibrosis was reduced. In addition, DCI improved the protein expression of MMP-9 and PPAR-γ in kidney tissue, which in turn inhibited NF-κB protein expression, as shown by immunohistochemistry and Western blotting. Our findings showed that DCI ameliorated the renal injury induced by diabetes by upregulating MMP-9 and PPAR-γ expression and downregulating NF-κB expression. We preliminarily concluded that the renal protective effect of DCI on diabetic mice may occurs through the MMP-9/NF-κB signalling pathway.

## INTRODUCTION

Diabetic nephropathy (DN) is a microvascular disease caused by diabetes that leads to glomerular sclerosis and is a serious progressive complication of diabetes [1, 2]. Hyperglycaemia, hypertension and genetic susceptibility are major risk factors for the development of diabetic nephropathy [3]. The prevention and treatment of DN is a long-term problem for diabetic patients [4].

Previous studies have shown that the main pathological features of early renal injury include glomerular and tubular hypertrophy, basement membrane thickening, and progressive extracellular matrix (ECM) accumulation in the mesangial area [5]. MMP-9 is the main physiological regulator of the ECM, and it can degrade various components of ECM and slow the progression of glomerular sclerosis [6]. PPAR-γ is a ligand-activated nuclear transcription factor that regulates many important physiological responses, including glucose metabolism, inflammation, renal interstitial fibrosis, and insulin resistance [7]. Activated and phosphorylated NF-κB subunits (P65 and P50) translocate to the nucleus and induce the expression of DN-related target genes [8]. It has been reported that regulating NF-κB activity can improve renal ECM accumulation and fibrosis [9]. The chemical name of DCI is 1,2,4-hexahydroxycyclohexane. In recent years, DCI has been widely used in clinical research. However, evidence of its physiological and therapeutic properties is steadily emerging. First, DCI was thought to be an insulin sensitizer and islet analogue that promoted insulin sensitivity and reduced blood glucose levels. We have found that DCI also plays a key role in regulation of steroid production and other important processes, such as intercellular adhesion, inflammation and oxidation. Therefore, DCI is used to treat various diseases in the clinic [10, 11]. However, the specific pathophysiological mechanism by which DCI improves diabetic kidney injury still needs to be further explored. In the present study, we aimed to investigate the effect of DCI on fibrosis in DN. Therefore, we selected the db/db mouse model to solve these problems. The regulatory effect of DCI on glucose metabolism was evaluated by examining blood glucose, blood lipids and renal function. Subsequently, morphological changes in the mouse kidney were observed under a microscope, and changes in the expression levels of fibrosis-related proteins were measured to preliminarily explore the renal protective mechanism of DCI.

## RESULTS

### Effects of DCI on the level of RBG in db/db mice

DCI RBG levels in db/db mice with type 2 diabetes (Table 1). Before administration, the RBG levels in db/db mice were significantly higher than those in db/m mice (*P* < 0.01), and the three groups were basically the same. After 6 weeks of administration, the RBG of HDCIG and LDCIG were decreased to some extent compared with those in the MCG. There was no significant difference between the HDCI and LDCI groups. Our findings indicated that DCI could effectively improve RBG levels in db/db mice.

**Table 1:** Effects of D-chiro-inositol on the level of blood glucose in db/db mice (mmol/L).

**Group**	**0 weeks**	**6 week**
NCG	7.22 ± 0.32	6.42 ± 0.47
MCG	20.56 ± 3.46**	27.58 ± 4.04**
HDCIG	20.52 ± 3.12	18.07 ± 4.26##
LDCIG	20.97 ± 3.31	19.12 ± 4.33##

Mean ± SD, *n* = 10; ^*^P < 0.05, ^**^p < 0.01 vs. NCG; ^#^*p* < 0.05, ^##^*p* < 0.01 vs. MCG.

### Effects of DCI on serum Cr, BUN and renal weight index in db/db mice

As shown in Table 2, serum Cr and BUN levels in the MCG were significantly higher than those in the NCG. Compared with those in the MCG, serum Cr and BUN levels results in the HDCIG and LDCIG were reduced, and the effect on the HDCIG was more significant than in the other groups. The kidney index of db/db mice was significantly lower than that of db/m mice (*P* < 0.01). The kidney index of each drug-administered group was lower than that of the MCG group, however there was no significant difference. Our experimental results showed that DCI effectively alleviated kidney damage caused by diabetes and significantly improved renal function.

**Table 2:** Effects of D-chiro-inositol on Cr, BUN and renal weight index in db/db mice.

**Group**	**Cr (μmol/L)**	**BUN (mmol/L)**	**Renal weight index (mg/g, %)**
NCG	37.93 ± 8.77	187.02 ± 22.40	1.24 ± 0.085
MCG	50.62 ± 6.74**	262.29 ± 32.20**	0.70 ± 0.084**
HDCIG	39.44 ± 8.04##	205.54 ± 23.62##	0.69 ± 0.099
LDCIG	43.22 ± 7.34#	223.24 ± 22.88##	0.65 ± 0.060

Mean ± SD, *n* = 10; ^*^p < 0.05, ^**^p < 0.01 vs. NCG; ^#^*p* < 0.05, ^##^*p* < 0.01 vs. MCG.

### Pathological staining of kidney tissues in db/db mice

**Figure 1:**
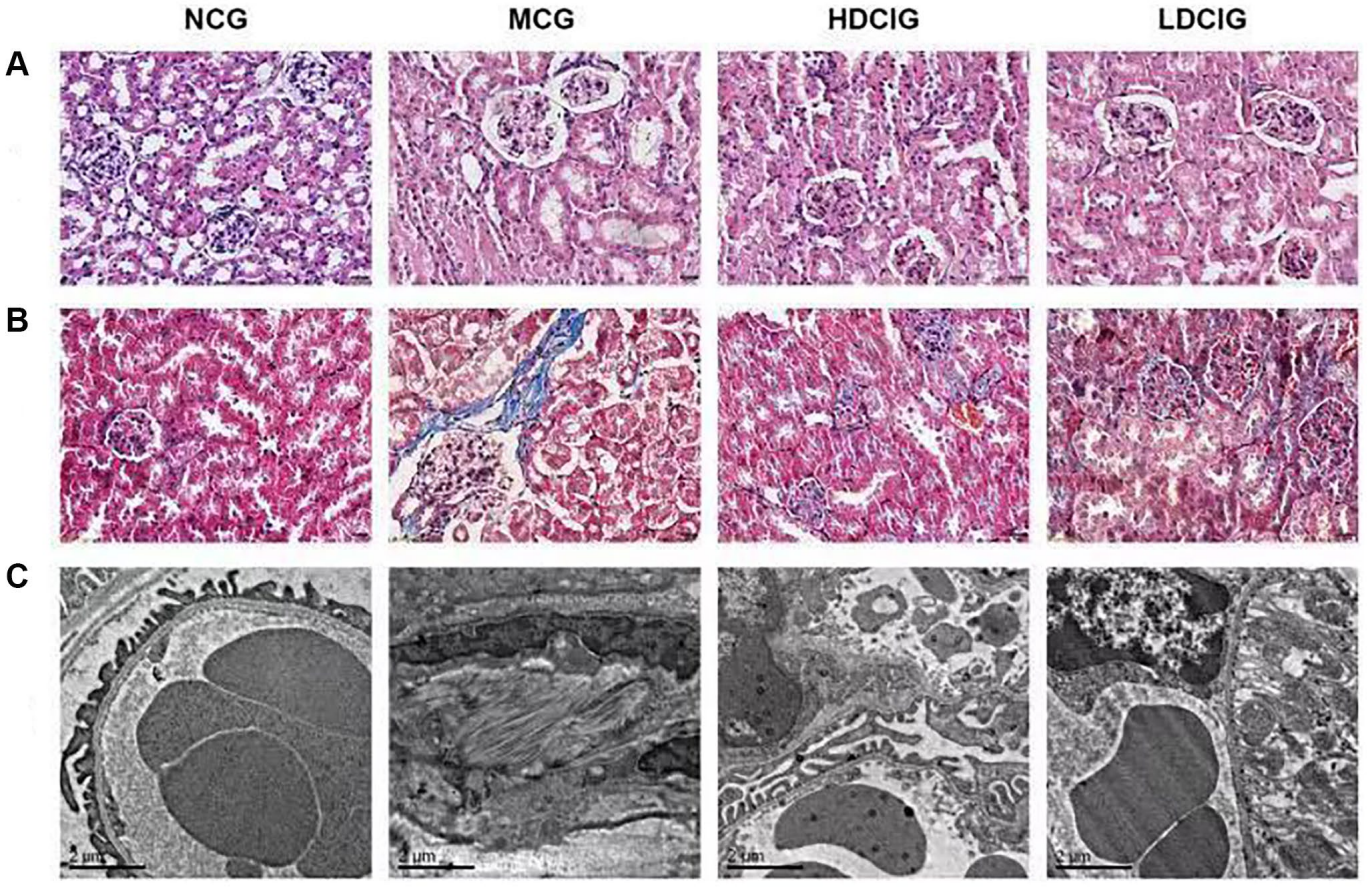
**Morphological structure of kidney tissues in db/db mice.** (**A**) HE staining; (**B**) Masson staining; (**C**) Electronic microscopy.

DCI significantly ameliorated histological changes in the kidney (Figure 1). HE staining showed that the nuclei in kidney tissue in the NCG were stained blue, the cytoplasm was stained pink, and the structure of the renal tubules and glomeruli was normal. The MCG had severe inflammatory cell infiltration and significant oedema of renal tubular epithelial cells. The HDCIG and LDCIG reduced renal tissue damage. Masson staining showed a significant increase in glomerular and renal interstitial fibrosis in the MCG, while that in the HDCIG and LDCIG was decreased to varying degrees.

As observed by transmission electron microscopy, glomerular mesangial matrix proliferated in the MCG, which was accompanied by podocyte fusion or disappearance and basement membrane thickening. Renal mesangial matrix hyperplasia and basement membrane thickening were attenuated in the HDCIG and LDCIG, and the podocyte structure was improved compared with that in the MCG.

### Immunohistochemical analysis of MMP-9, PPAR-γ and NF-κB protein expression

**Figure 2:**
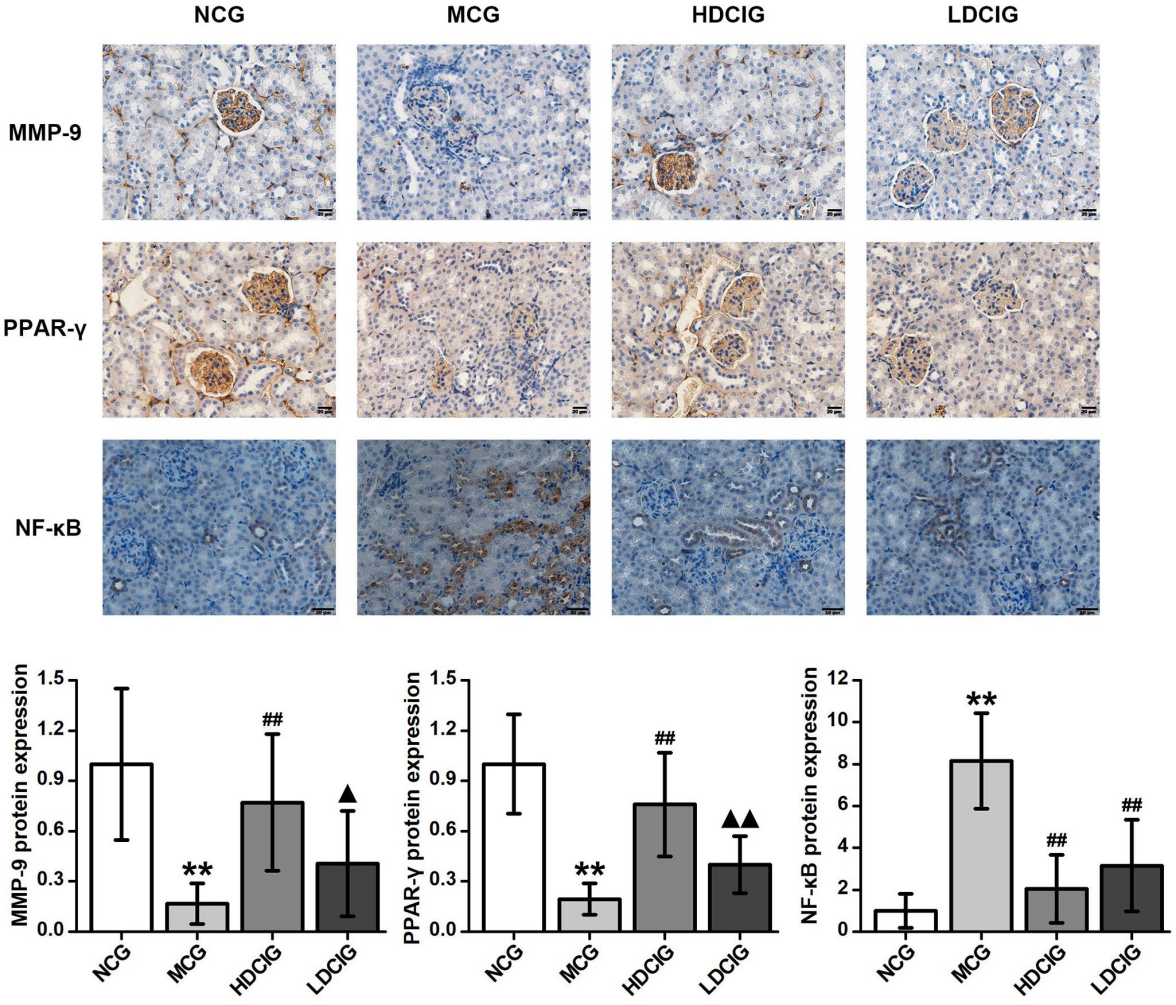
**Immunohistochemistry determination of the relative protein expression in the kidney tissues of db/db mice (*n* = 4).** The results obtained for the different groups were normalized to those reported in the normal control group (NCG). All values are expressed as the means ± SD. ^*^p < 0.05, ^**^p < 0.01 vs. NCG mice; ^#^*p* < 0.05. ^##^*p* < 0.01 vs. MCG mice. ^▲^*p* < 0.05, ^▲▲^*p* < 0.01 vs. HDCIG mice.

The immunohistochemical staining analysis are shown in Figure 2. MMP-9 and PPAR-γ proteins are expressed on mouse kidney glomerular cells. These proteins were highly expressed in the kidney tissue in the NCG, and the positive staining was brownish yellow and evenly distributed. The protein expression of MMP-9 and PPAR-γ in the MCG was lower than that in the NCG (*P* < 0.01). Moreover, the expression levels of MMP-9 and PPAR-γ in the HDCIG were significantly higher than those in the MCG. The NF-κB protein was expressed in the cytoplasm of tubular cells. The protein expression of NF-κB protein was lower in the NCG and higher in the MCG. The expression levels of NF-κB protein in the HDCIG and LDCIG were decreased, but there was no significant difference between the two groups.

### Western-blot analysis of MMP-9, PPAR-γ and NF-κB protein expression

**Figure 3:**
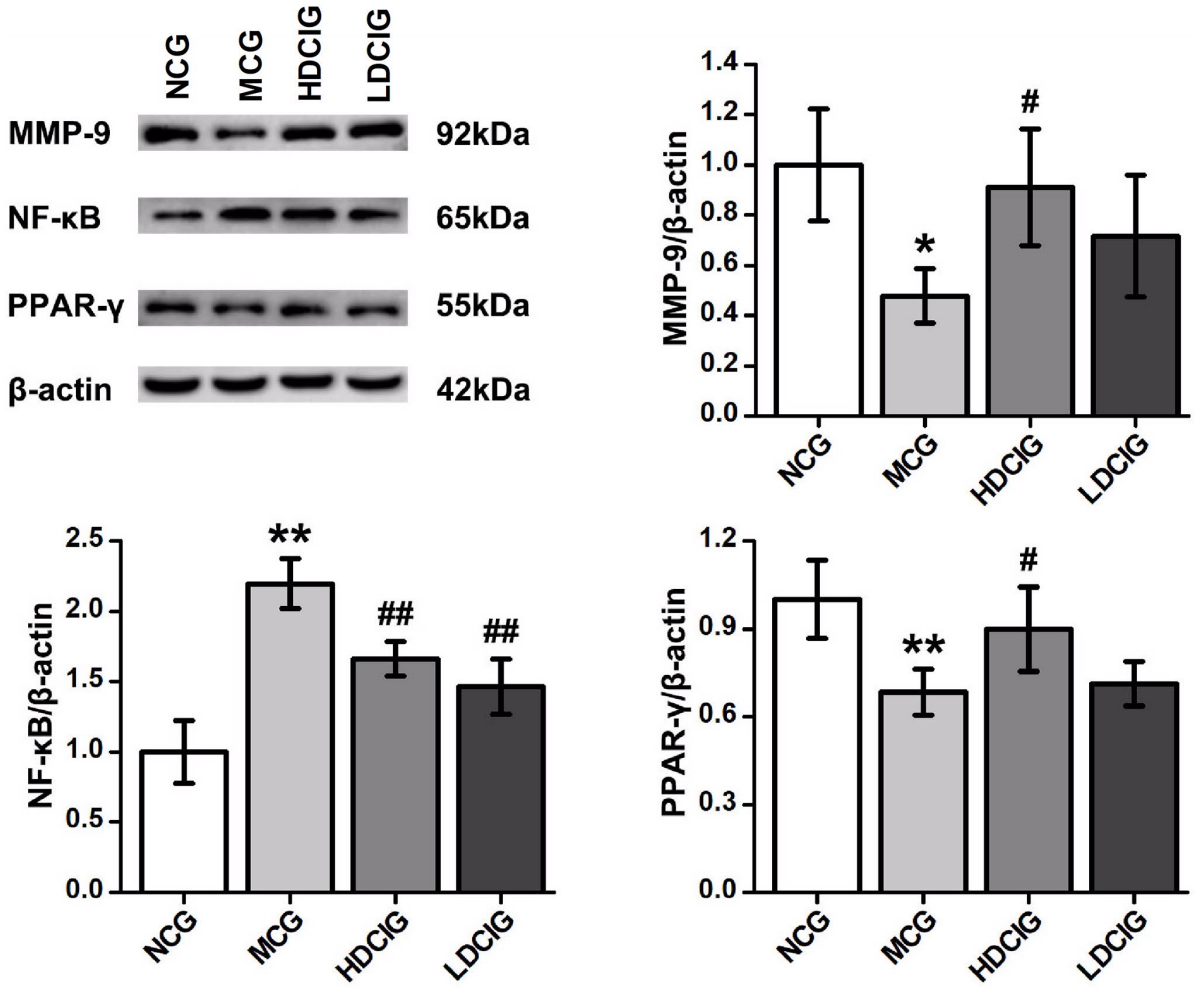
**Effects of D-chiro-inositol on relative protein expression in the kidney tissues of db/db mice (*n* = 3).** The results obtained for the different groups were normalized to those reported in the normal control group (NCG). All values are expressed as the means ± SD. ^*^p < 0.05, ^**^p < 0.01 vs. NCG mice; ^#^*p* < 0.05. ^##^*p* < 0.01 vs. MCG mice. ^**▲**^*p* < 0.05, ^**▲▲**^*p* < 0.01 vs. HDCIG mice.

The results showed that the expression levels of MMP-9 and PPAR-γ protein in the MCG were lower than those in NCG (Figure 3). The protein expression of MMP-9 and PPAR-γ in the HDCIG was higher than that in the MCG. The expression of NF-κB protein in MCG was significantly higher than that in the NCG. Compared with those in the MCG, the protein expression levels of NF-κB in the HDCIG and LDCIG were decreased. These findings demonstrate the unique ability of DCI to mitigate renal fibrosis by acting on pathophysiological renal fibrosis events, as well as the proteins associated with renal fibrosis.

## DISCUSSION

DN is a serious complication associated with the development of diabetes and a major cause of end-stage renal disease [12]. The pathogenesis of DN is closely related to a variety of factors, including glucose and lipid metabolism disorders, hemodynamic abnormalities, oxidative stress, cytokines and inflammatory factors [13]. Many basic research studies have proven that renal fibrosis is the most important process in the progression of multiple chronic kidney diseases (including DN) to end-stage renal disease [14–16].

Renal fibrosis is dominated by tubulointerstitial fibrosis, which may manifest as glomerular sclerosis. Db/db mice are an ideal animal model that spontaneously develops type 2 diabetes [17]. This study investigated the effects of DCI on kidney tissue in db/db mice.

A previous study showed that DCI reduced blood glucose levels in db/db mice and protected the liver [18]. Combined with the blood glucose results of this experiment study, these results show that DCI has a good hypoglycaemic glycemic effect and improves the body’s glucose metabolism. Serum Cr and BUN levels are two important indicators for when clinically assessing kidney damage [19]. The serum results showed that DCI reduced the content levels of Cr and BUN in the serum of db/db mice and promoted glomerular filtration. Additionally, pathological staining showed that DCI reduced the proliferation of glomerular mesangial matrix in db/db mice and reduced the degree of renal fibrosis. This finding indicates that DCI alleviates kidney damage in db/db mice.

The MMP family is a major factor in the degradation of various ECM components [20], and of in this family, MMP-9 is a key enzyme that regulates ECM degradation [21], and is closely related to the occurrence and development of DN. Abnormal expression of MMP-9 is involved in proteinuria-related kidney diseases, including focal segmental glomerulosclerosis and anti-glomerular basement membrane inflammation [6]. Under high glucose stimulation, the gene expression of multiple mesangial matrix components is upregulated, and ECM synthesis is increased. In addition, MMP-9 expression is affected, and ECM degradation is reduced, promoting the process of renal fibrosis. Therefore, it is important to maintain the normal level of MMP-9. The transcription factor NF-κB is involved in the transcriptional regulation of many adhesion molecules and inflammatory cytokines, such as TNF-α, IL-10, and ICAM-1 [22, 23], so and increased levels of NF-κB leads to inflammatory reactions [24]. PPAR-γ plays an important role in improving insulin resistance, promoting glucose metabolism, lowering blood pressure, inhibiting fat accumulation in the kidney, increasing adiponectin expression, protecting podocytes, inhibiting renal interstitial fibrosis, and exerting anti-inflammatory and antioxidant effects [25–27]. Studies have shown that PPAR-γ inhibits NF-κB signalling by inhibiting the transcriptional activity of NF-κB, thereby inhibiting the expression of inflammatory factors associated with TNF-α [28]. In the current study, we found that DCI certainly enhanced the expression of PPAR-γ and MMP-9, decreased NF-κB activity, and reduced renal fibrosis in mice. These results suggested that enhancing PPAR-γ protein expression ameliorated fibrosis at least in part through by modulating the activity of the NF-κB and the expression of MMP-9. Therefore, based on our results, we speculate hypothesize that DCI protects the kidney by enhancing the expression of PPAR-γ and possibly alleviating renal fibrosis through the NF-κB/ MMP-9 signalling pathway.

## CONCLUSIONS

In summary, DCI reduces blood glucose and kidney tissue damage in db/db mice. This outcome may be related to increases in the protein expression of MMP-9 and PPAR-γ and the inhibition of NF-κB protein expression in the kidney tissue of db/db mice. However, the mechanism of DCI may involve multiple targets and multiple pathways, and further research is needed.

## MATERIALS AND METHODS

### Animal care

Male db/db mice (8 weeks, 29–35 g) and male db/m mice (8 weeks, 18–22 g) were purchased from Changzhou Cavens Laboratory Animal Co. Ltd. (Changzhou, China) (licence key: SCXK (Su) 2016-0010). Mouse granule feedstuff treated ^60^Co radiation was purchased from Nanjing Beisifu Feed Co. Ltd. (Nanjing, China). The animal experiments were performed in specific-pathogen-free barrier laboratory at the Experimental Animal Centre of North China University of Science and Technology (Tangshan, China). All procedures for animal experiments were approved by the Animal Ethics Committee of North China University of Science and Technology, according to the guidelines established by the European Union (Directive 2010/63/EU for animal experiments) and the National Institute of Health of the USA (NIH Publications No. 8023, revised 1978).

### Grouping and administration

After adaptive feeding for 1 week, blood was drawn from the tails of the mice to determine the levels of blood glucose using a blood glucose metre (Roche, USA). Ten db/m mice were selected to establish the normal control group (NCG). Based on the RBG levels, the 30 db/db mice were randomly divided into three groups received different treatments: high-dose DCI group (HDCIG), low-dose DCI group (LDCIG) and model control group (MCG).

The mice in the HDCIG and LDCIG mice received DCI (purity: >98.0 %, I0632, TCI, Japan) at a dose of 70 mg/kg/d and 35 mg/kg/d, respectively. The NCG and MCG mice received an equal volume of pure water. All treatments were administered at 9 a.m. every day, and the duration of the entire experiment was 6 weeks.

### Laboratory analyses

RBG measurements were conducted in each group at 0 and 6 weeks used tail vein blood after intragastric administration. Twenty-four hours after the last administration, blood samples were collected from the eye socket, and the serum was separated. Cr and BUN levels were measured using Assayan assay kit (Nanjing Jiancheng Bioengineering Institute, C011-2-1/1/C013-2-1, China) according to the Kit instructions.

### Histopathological

After being weighed, 1 mm^3^ kidney tissue blocks were cut at the renal cortex, fixed with 0.025% glutaraldehyde solution for 12 h, incubated with phosphate buffer -buffered saline (PBS) for 8 h, and incubated with 0.01% osmic acid for 2 h. Then, the samples were rinsed and dehydrated, embedded with Epon 812, sliced (70 nm), and stained with lead citrate. Finally, the samples were observed by transmission electron microscopy (H-7650, Hitachi Limited, Japan).

The kidney tissue was fixed in 4% paraformaldehyde for 72 h, and subsequently subjected to conventional paraffin embedding. Then, the kidney tissue was cut into 3.5-μm-thick sections. After HE staining and Masson staining, the histological structure of the kidney tissue was observed under a BX50 microscope (Olympus, Japan).

### Immunohistochemistry

Immunohistochemistry was performed using a universal two-step test kit (ZSGB-BIO, PV-9000, China). Paraffin-embedded sections of kidney tissues were dewaxed, hydrated, and boiled to repair antigens through ethylenediaminetetraacetic acid (EDTA) high-pressure heating. Following a wash with PBS, the sections were soaked with serum (ZSGB-BIO, ZLI-9022, China) and incubated with the following primary antibodies: anti-MMP-9 (E-11) (1:300, Santa Cruz, sc393859, USA), anti-PPAR-γ (1:200, Santa Cruz, sc7273, USA) and anti-NF-κB (1:200, Santa Cruz, sc8008, USA). After being incubated at 4°C overnight, the sections were incubated with the secondary antibody provided by the kit at 37°C. After being washed with PBS, the sections were dyed using 3,3′-diaminobenzidine (ZSGB-BIO, ZLI-9018, China) for 50 s. The nucleus was stained with hematoxylin for 1 min. In the negative control group, PBS was used instead of primary antibodies in the experiments. Finally, the positive proteins were colored brownish yellow, and the expression and distribution of indicated proteins were observed under a light microscope. The average integral absorbance was determined using Image-Pro Plus 6.0 software.

### Western blot analysis

The kidney tissue was homogenized in protein lysate buffer (Leagene, PS0013, China). The supernatant was harvested after centrifugation at 4°C, and the protein was quantified using a bicinchoninic acid assay kit (Leagene, PT0006, China). The proteins were separated by gradient sodium dodecyl sulfate–polyacrylamide gel electrophoresis (Leagene, PE0017, China) and electrophoretically transferred to polyvinylidene difluoride blotting membranes (GE, 10600023, USA). The membranes were blocked with 5% non-fat dry milk for 2 h, and subsequently incubated with primary antibodies overnight at 4°C. The primary antibodies used were as follows: anti- MMP-9 antibody (1:500, Santa Cruz, sc393859), anti- PPAR-γ (1:500, Santa Cruz, sc7273), anti-NF-κB (1:500, SANTA, sc8008, USA) and anti-β-actin (1:5,000, Bioworld, BS6007M, USA). After being washed with TBST (Solarbio, T1081, China), the membranes were incubated with anti-mouse HRP labelled secondary antibodies (1:5,000, SeraCare, 5450-0011, USA). Signal quantification was performed using an Odyssey Infrared Imaging System (Li-COR, USA). The protein bands were normalized to the β-actin band in each sample.

### Statistical analysis

Statistical analysis was performed using SPSS 19.0 software, and the experimental data are expressed as the means ± standard deviation (SD). Statistical significance was determined using one-way analysis of variance, followed by a least significant difference test for multiple comparisons. *P* < 0.05 denoted statistical significance.

### Ethics statement

The experiments were approved by the Animal Ethics Welfare Committee of North China University of Science and Technology.
